# Complete Genome Sequence and Characterization of Linezolid-Resistant *Enterococcus faecalis* Clinical Isolate KUB3006 Carrying a *cfr*(B)-Transposon on Its Chromosome and *optrA*-Plasmid

**DOI:** 10.3389/fmicb.2018.02576

**Published:** 2018-10-25

**Authors:** Makoto Kuroda, Tsuyoshi Sekizuka, Hidehito Matsui, Katsunori Suzuki, Hiroyuki Seki, Mitsumasa Saito, Hideaki Hanaki

**Affiliations:** ^1^Pathogen Genomics Center, National Institute of Infectious Diseases, Tokyo, Japan; ^2^Infection Control Research Center, Kitasato University, Tokyo, Japan; ^3^Division of Infection Control and Prevention, University of Occupational and Environmental Health, Kitakyusyu, Japan; ^4^Department of Microbiology, School of Medicine, University of Occupational and Environmental Health, Kitakyusyu, Japan

**Keywords:** linezolid, *Enterococcus*, *cfr*(B), Tn*6218*, *optrA*

## Abstract

Linezolid (LZD) has become one of the most important antimicrobial agents for infections caused by gram-positive bacteria, including those caused by *Enterococcus* species. LZD-resistant (LR) genetic features include mutations in 23S rRNA/ribosomal proteins, a plasmid-borne 23S rRNA methyltransferase gene *cfr*, and ribosomal protection genes (*optrA* and *poxtA*). Recently, a *cfr* gene variant, *cfr*(B), was identified in a Tn*6218*-like transposon (Tn) in a *Clostridioides difficile* isolate. Here, we isolated an LR *Enterococcus faecalis* clinical isolate, KUB3006, from a urine specimen of a patient with urinary tract infection during hospitalization in 2017. Comparative and whole-genome analyses were performed to characterize the genetic features and overall antimicrobial resistance genes in *E. faecalis* isolate KUB3006. Complete genome sequencing of KUB3006 revealed that it carried *cfr*(B) on a chromosomal Tn*6218*-like element. Surprisingly, this Tn*6218*-like element was almost (99%) identical to that of *C. difficile* Ox3196, which was isolated from a human in the UK in 2012, and to that of *Enterococcus faecium* 5_Efcm_HA-NL, which was isolated from a human in the Netherlands in 2012. An additional oxazolidinone and phenicol resistance gene, *optrA*, was also identified on a plasmid. KUB3006 is sequence type (ST) 729, suggesting that it is a minor ST that has not been reported previously and is unlikely to be a high-risk *E. faecalis* lineage. In summary, LR *E. faecalis* KUB3006 possesses a notable Tn*6218*-like-borne *cfr*(B) and a plasmid-borne *optrA*. This finding raises further concerns regarding the potential declining effectiveness of LZD treatment in the future.

## Introduction

Since gaining regulatory approval in 2000 for clinical use, linezolid (LZD) has become one of the most important antimicrobial agents for infections caused by gram-positive bacteria, including methicillin-resistant *Staphylococcus aureus* (MRSA) and vancomycin (VCM)-resistant enterococci (Zahedi Bialvaei et al., 2017). *Enterococcus faecalis* is a lactic acid-producing gram-positive bacterium that is commonly found in the intestinal tracts of humans and animals and is implicated in several fatal clinical infections, such as bacteremia and infective endocarditis (Dahl and Bruun, 2013; Falcone et al., 2015; Beganovic et al., 2018).

LZD-resistant (LR) isolates generally exhibit alterations in the central loop of domain V in the 23S rRNA in the bacterial ribosome. In enterococci, the G_2576_T (*Escherichia coli* numbering) mutation in the 23S rRNA gene(s) has been the predominant cause of the loss of susceptibility to LZD (Kloss et al., 1999), and additional mutations in the L3/L4 ribosomal proteins have also been shown to cause decreased susceptibility to LZD (Mendes et al., 2014).

In addition, a plasmid-borne chloramphenicol-florfenicol resistance gene, *cfr*, was identified in a *Staphylococcus sciuri* isolate obtained from the nasal swab of a calf (Schwarz et al., 2000). This plasmid-borne LR gene has been also identified in a human clinical MRSA isolate (Toh et al., 2007). Cfr methyltransferase, which mediates the transfer of methyl residues on adenine 2503 in 23S rRNA (Kehrenberg et al., 2005), can mediate the PhLOPS_A_ phenotype (resistance to phenicols, lincosamides, oxazolidinones, pleuromutilins, and streptogramin A compounds) (Long et al., 2006). The *cfr* gene has been documented in a variety of bacterial isolates and first emerged in coagulase-negative staphylococci (CNS) (Witte and Cuny, 2011). LR *E. faecalis* carrying *cfr* was first described in an animal isolate in 2011 from China (Liu et al., 2012); subsequently, clinical LR *E. faecalis* isolate was identified from a patient subjected to prolonged antimicrobial therapy in Thailand in 2010 (Diaz et al., 2012). Further studies have suggested that livestock-associated CNS (He et al., 2014; Schoenfelder et al., 2017), MRSA (Li et al., 2017), and *Enterococcus* spp. (Torres et al., 2018) have disseminated and harbor a significant resistance gene pool, including *cfr*, in livestock environments.

The plasmid-mediated LR determinant *optrA*, encoding the ATP-binding cassette F (ABC-F) family protein, was first characterized and identified in *E. faecalis* and *E. faecium* from food-producing animals and from humans in China in 2009 (Wang et al., 2015). ABC-F proteins have been classified into three groups based on their antibiotic resistance: (i) Msr homologs, resistant to macrolides and streptogramin B; (ii) Vga/Lsa/Sal homologs, resistant to lincosamides, pleuromutilins, and streptogramin A; and (iii) OptrA homologs, resistant to phenicols and oxazolidinones (Sharkey et al., 2016). Unlike other ABC transporters, these ABC-F proteins lack the transmembrane domain characteristic to transporters and are believed to confer antibiotic resistance via a ribosomal protection mechanism by interacting with the ribosome and displacing the bound drug (Sharkey et al., 2016). A cryo-EM structural analysis demonstrated a universal resistance mechanism in which ABC-F protein binding leads to ribosomal conformational changes, resulting in the release of the antibiotic (Su et al., 2018). Further epidemiological study for *optrA* dissemination suggested that nationwide surveillance for *optrA*-positive LR *Enterococcus* isolates in China showed a marked increase in detection from 0.4 to 3.9% during the 10-year period (2004–2014) (Cui et al., 2016), and the review summarized the *optrA*-positive LR *Enterococcus* isolates from animal origins and environment (Torres et al., 2018).

Recently, the newly identified *poxtA* gene in the MRSA AOUC-0915 strain was found to encode an OptrA homolog. The expression of *poxtA* in *E. coli, S. aureus*, and *E. faecalis* results in a decrease in susceptibility to phenicols, oxazolidinones, and tetracyclines (Antonelli et al., 2018).

Two clinical surveillance programs have monitored LZD susceptibility among clinically significant isolates. The global Zyvox Annual Appraisal of Potency and Spectrum (ZAAPS) program, comprising medical centers in 32 ex-USA countries, reported the continued long-term and stable *in vitro* potency of LZD against staphylococci and *Enterococcus faecium* clinical isolates in 2015 (Pfaller et al., 2017b). However, a limited number of isolates exhibited mutations in the 23S rRNA gene and/or L3/L4-encoding proteins, in addition to plasmid-mediated resistance determinants (*cfr* and *optrA*), leading to a decreased susceptibility to LZD [A minimum inhibitory concentration (MIC) of ≥8 μg/mL is considered “resistant” by CLSI M100-S28, while a MIC of >4 mg/L is considered “resistant” by EUCAST]. The USA Linezolid Experience and Accurate Determination of Resistance (LEADER) program has reported that the overall LR rate remained a modest 1% in enterococci from 2011 to 2015 (Pfaller et al., 2017a), but clonal dissemination of LR strains has been suggested in staphylococci and *E. faecium* clinical isolates based on pulsed-field gel electrophoresis (PFGE) profile analysis.

Recently, a *cfr* gene variant, *cfr*(B), was identified from *Clostridioides* (formerly *Clostridium* or *Peptoclostridium*) *difficile* isolates (Marin et al., 2015). Further investigation in the United States under the SENTRY antimicrobial surveillance program suggested that *cfr*(B)-positive *E. faecium* was found among human clinical isolates (Deshpande et al., 2015). An increasing number of LR *E. faecium* clinical isolates from <1% in 2008 to >9% in 2014 in Germany has caused a concern (Klare et al., 2015). Moreover, *cfr*(B) from *E. faecium* isolates in Germany was acquired in a plasmid-mediated manner, following *cfr*(B) plasmid integration on the chromosome in some isolates (Bender et al., 2016). In contrast to the plasmid-borne *cfr* and *cfr*(B) genes, the *cfr*(B) gene was observed to be chromosomally located and embedded in a Tn*6218*-like transposon (Tn) in the *C. difficile* strains Ox2167 and Ox3196 (Deshpande et al., 2015).

Various mobile genetic elements (MGE) have been shown to contribute to the acquisition of 23S rRNA methyltransferases [*cfr* and *cfr*(B)] and ABC-F protein (OptrA) for their dissemination in clinically relevant gram-positive pathogens such as enterococci and streptococci (Sadowy, 2018). A comprehensive molecular investigation in both humans and veterinary subjects may be required to preserve this pivotal antibiotic for gram-positive bacterial infections. In this study, we determined the complete genome sequence of *cfr*(B)-positive LR *E. faecalis* KUB3006 and the plasmid carrying *optrA*, which is the first report of a Tn*6218*-like-embedded *cfr*(B)-positive *E. faecalis* clinical isolate.

## Materials and Methods

### Ethics Approval and Consent to Participate

The study protocol was approved by the National Institute of Infectious Diseases in Japan (Approval No. 677) and was conducted in accordance with the tenets of the Declaration of Helsinki. Written informed consent was obtained from the patient for the publication of this manuscript. The consent form is held by the authors’ institution and is available for review.

### Bacterial Strains

*Enterococcus faecalis* strain KUB3006 was isolated from the midstream urine of a 67-year-old patient during hospitalization on May 2nd, 2017. The patient was suffering from collagen disease under the treatment of steroid and other immunosuppressive agents; however, such immune-compromised status increased the susceptibility to successive infection. The MIC for all of the following antimicrobials was determined by the broth-dilution method using the CLSI criteria (M100-S28, 2018): LZD, linezolid; VCM, vancomycin; TEIC, teicoplanin; ABK, arbekacin; TOB, tobramycin; LVFX, levofloxacin; AMP, ampicillin; IPM, imipenem; EM, erythromycin; SPM, spectinomycin; CLDM, clindamycin; CP, chloramphenicol.

### Whole-Genome Sequence Analysis

Genomic DNA from *E. faecalis* was purified as follows. Bacterial cells were collected from a 5-mL overnight culture suspended in 500 μL TE10 [10 mM Tris (pH 8.0) and 10 mM EDTA]. The cell suspension was supplemented with 500 μL phenol/chloroform, followed by bead-beating for 10 min by vortexing in ZR BashingBead lysis tubes (Zymoresearch, Irvine, CA, United States) attached to a vortex adapter (Mo Bio Laboratories, Qiagen, Carlsbad, CA, United States). After centrifugation at 10,000 × *g* for 5 min, the upper phase was further purified using a Qiagen DNA purification kit (Qiagen). A DNA-seq library (approximately 0.5-kb inserts) was constructed using a QIAseq FX DNA Library Kit (Qiagen). Whole-genome sequencing was performed using the Illumina NextSeq 500 platform with the 300-cycle NextSeq 500 Reagent Kit v2 with paired-end read sequencing (2 × 150-mer; median coverage: 268×).

The complete genome sequences of the strain was determined using the long-read sequencing method of the PacBio Sequel sequencer [Sequel SMRT Cell 1M v2 (4/tray); Sequel Sequencing Kit v2.1; insert size, approximately 10 kb]. Purified genomic DNA (∼200 ng) was used to prepare a SMRTbell library using a SMRTbell Template Prep Kit 1.0 (PacBio, Menlo Park, CA, United States) with barcoded adaptors according to the manufacturer’s instructions.

Sequencing data were produced with more than 100-fold coverage and assembled using the following programs: Canu version 1.4 (Koren et al., 2017), Minimap version 0.2-r124 (Li, 2016), Racon version 1.1.0 (Vaser et al., 2017), and Circlator version 1.5.3 (Hunt et al., 2015). Error correction of tentative complete circular sequences was performed using Pilon version 1.18 with Illumina short reads (Walker et al., 2014). Annotation was performed in Prokka version 1.11 (Seemann, 2014), InterPro v49.0 (Finn et al., 2017), and NCBI-BLASTP/BLASTX.

Circular representations of complete genomic sequences were visualized using the GView server (Petkau et al., 2010). Antimicrobial resistance (AMR) genes were identified by homology searching against the ResFinder database (Zankari et al., 2012). Multilocus sequence typing (MLST) was performed using SRST2 (Inouye et al., 2014). Virulence factors for *Enterococcus* spp. were predicted using VirulenceFinder analysis (Kleinheinz et al., 2014).

### Comparative Genome Sequence Analysis

All publicly available draft genome sequences of *E. faecalis* strains were retrieved (>2,000 strains with least 40× read coverage) and compared by using bwaMEM to map reads to the *E. faecalis* KUB3006 complete genome sequence (GenBank ID: AP018538) as a reference. Repeat regions were identified and excluded from further core-genome phylogenetic analysis using NUCmer (Kurtz et al., 2004), as these single-nucleotide variation (SNV) sites are considered unreliable. The core genome SNV analysis was performed using the maximum likelihood phylogenetic method with FastTree v2.1.10. Comparative Tn sequence analysis was performed with a BLASTN search (≥80% nt identity), followed by visualization using Easyfig v2.2.2 (Sullivan et al., 2011).

The *cfr*(B) gene SNV analysis was conducted using the median joining network method with PopART (Leigh and Bryant, 2015).

### Nucleotide Sequence Accession Numbers

The complete genomic sequences and annotations of *E. faecalis* strain KUB-3006 were deposited in a public database DDBJ: chromosome (GenBank ID: AP018538); pKUB3006-1 (GenBank ID: AP018539); pKUB3006-2 (GenBank ID: AP018540); pKUB3006-3 (GenBank ID: AP018541); and pKUB3006-4 (GenBank ID: AP018542). The short- and long-read DNA sequences have been deposited in the DDBJ Sequence Read Archive under accession number DRA006641 (BioProject: PRJDB6823, BioSample: SAMD00113788-SAMD00113789, and Experiment: DRX11916-DRX119165).

## Results

### Antimicrobial Susceptibility Testing

Compared with the *E. faecalis* type strain ATCC 29212 as a standard, *E. faecalis* KUB3006 showed resistance to LZD, ABK, TOB, LVFX, EM, SPM, CLDM, and CP, but not to VCM, TEIC, or β-lactams (AMP and IPM). This suggests that KUB3006 exhibits susceptibility to glycopeptides (VCM and TEIC) but reduced susceptibility to LZD (MIC: 16 μg/mL) (Table 1).

**Table 1:** Antimicrobial susceptibility test (MIC, μg/mL).

Antimicrobial agents	*E. faecalis* KUB3006	*E. faecalis* ATCC 29212
LZD	16	2
VCM	2	2
TEIC	0.5	0.25
ABK	>128	32
TOB	>128	16
LVFX	>128	1
AMP	2	1
IPM	2	0.5
EM	>64	2
SPM	>128	1
CLDM	>128	16
CP	64	8


*LZD, linezolid; VCM, vancomycin; TEIC, teicoplanin; ABK, arbekacin; TOB, tobramycin; LVFX, levofloxacin; AMP, ampicillin; IPM, imipenem; EM, erythromycin; SPM, spectinomycin; CLDM, clindamycin; CP, chloramphenicol.*

### Basic Genome Information for KUB3006

**FIGURE 1:**
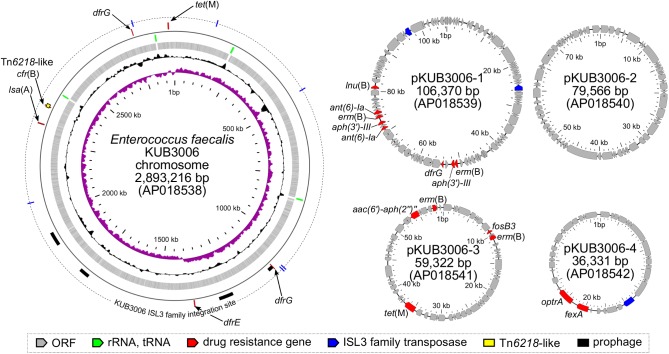
Circular representation of the LR *E. faecalis* KUB3006 genome (chromosome and four plasmids). Moving inward in the chromosome circular map, slots 1–4 (slot 1, GC skew; slot 2, GC content; slot 3, open reading frames; slot 4, RNAs), slot 5 (prophage, AMR gene), and slot 6 (insertion sites of IS L3 family in KUB3006).

Basic information related to the complete genome sequence of *E. faecalis* strain KUB3006 is shown in Figure 1. To characterize the LZD resistance of this strain, potential mutations in the 23S rRNA genes, ribosomal protein genes (*rplC, rplD*, and *rplV*), and *cfr* 23S rRNA methylase gene were investigated, but no notable genetic features were identified. However, KUB3006 possesses the *cfr* variant *cfr*(B) on the chromosome, as well as four plasmids carrying multiple AMR genes, including *optrA* on pKUB3006-4 (36.3 kb) (Figure 1).

Genome analysis of the complete chromosomal DNA using SRST2 indicated that KUB3006 is classified as sequence type (ST) 729. VirulenceFinder analysis (Kleinheinz et al., 2014) demonstrated that KUB3006 carries multiple cell-adhesion properties [biofilm formation proteins (*ebpA, ebpC, fsrB*); adhesin to collagen (*ace*); an internalin-like Enterococcal leucine-rich protein A (*elrA*)] and cell-damaging factors [zinc-metalloprotease (*gelE*) for host collagen, fibrinogen, and fibrin; hyaluronidase (*hylA* and *hylB*)] (Table 2). In addition, multiple sex pheromones (*camE, cOB1, cAD1*, and *cCF10*) on its chromosome and two aggregation substances (*agg*) in two plasmids (pKUB3006-1 and pKUB3006-2) were found for conjugative transfer of plasmid (Table 2).

**Table 2:** Prediction of virulence factors in LR *E. faecalis* KUB3006 by VirulenceFinder.

Virulence factor	Identity (%)	Query/Template length (nt)	*E. faecalis* KUB3006 genome (GenBank ID)	Position in genome	Protein function
**Adhesin and aggregation**					
*ace*	95.71	2166/2166	Chromosome (AP018538.1)	903926..906091	Collagen adhesin precursor
*ebpA*	99.58	3312/3312	Chromosome (AP018538.1)	891588..894899	Endocarditis and biofilm-associated pili for adherence to fibrinogen
*ebpC*	99.58	1884/1884	Chromosome (AP018538.1)	896330..898213	Endocarditis and biofilm-associated pili for adherence to fibrinogen
*efaAfs*	100	927/927	Chromosome (AP018538.1)	1820636..1821562	*Enterococcus faecalis* endocarditis antigen
*ElrA*	99.91	2172/2172	Chromosome (AP018538.1)	2275786..2277957	Enterococcal Leucine Rich protein A, an internalin-like protein
*fsrB*	99.59	729/729	Chromosome (AP018538.1)	1629581..1630309	Biofilm formation
*SrtA*	99.18	735/735	Chromosome (AP018538.1)	2570541..2571275	Sortase
**Degrading enzyme**					
*gelE*	100	1530/1530	Chromosome (AP018538.1)	1626474..1628003	Gelatinase
*hylA*	99.33	3266/3264	Chromosome (AP018538.1)	2547513..2550777	Hyaluronidase
*hylB*	99.4	3015/3015	Chromosome (AP018538.1)	601784..604798	Hyaluronidase
*tpx*	99.22	510/510	Chromosome (AP018538.1)	2470693..2471202	Lipid hydroperoxide peroxidase
**Sex pheromone and aggregation**					
*camE*	99.4	501/501	Chromosome (AP018538.1)	1159865..1160365	Sex pheromone cAM373
*cOB1*	99.39	819/819	Chromosome (AP018538.1)	2128279..2129097	Sex pheromone cOB1
*cad*	99.78	930/930	Chromosome (AP018538.1)	2785668..2786597	Sex pheromone cAD1
*cCF10*	99.76	828/828	Chromosome (AP018538.1)	2891232..2892059	Sex pheromone cCF10
*agg*	95.64	3920/3918	Plasmid pKUB3006-1 (AP018539.1)	15542..19459	Aggregation substance
*agg*	93.52	3918/3906	Plasmid pKUB3006-2 (AP018540.1)	9883..13779	Aggregation substance


### Core Genome Phylogenetic Analysis of KUB3006

**FIGURE 2:**
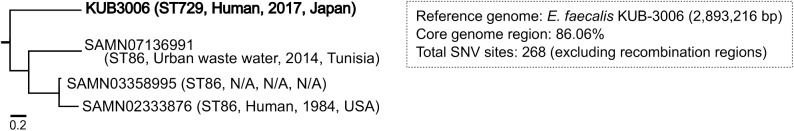
Maximum likelihood core genome phylogeny of *E. faecalis* KUB3006. The core genome phylogeny of *E. faecalis* isolates, including KUB3006, using the maximum-likelihood method.

To trace the potential source of the KUB3006 strain, we performed core genome phylogenetic analysis using > 2,000 publicly available *E. faecalis* genome sequences, including draft genomes. Among the core-genome sequences, a total of 268 SNVs were identified with the more relative *E. faecalis* three strains (Figure 2). The phylogeny indicated that KUB3006 belonged to a similar lineage as ST86 *E. faecalis* strains carrying *optrA-*positive plasmid (pAF379, GenBank assembly ID: GCA_002220885.1) isolated from urban wastewater in Tunisia and human clinical specimens isolated in 1984 in the United States (Figure 2). However, this phylogeny-based analysis did not reveal the source of KUB3006, indicating that further genome sequences are required to determine a common source of the strain.

### *cfr*(B) in the Tn*6218*-Like Tn

**FIGURE 3:**
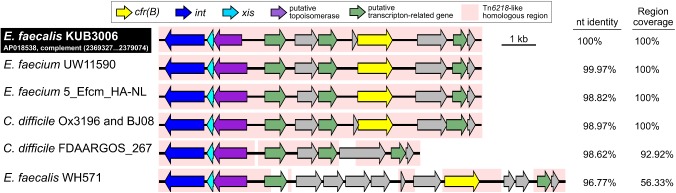
Representation of the structure of *cfr*(B)-positive Tn*6218*-related Tns. Structural organization of a Tn*6218*-like Tn carrying *cfr*(B) in *E. faecalis* KUB3006 and the nucleotide identity compared with those of related Tns in *C. difficile* clinical isolate*s* and *Enterococcus* species isolates.

The *cfr*(B) gene was located in the Tn*6218*-like Tn element (2,369,327–2,379,074 nt on the KUB3006 chromosome in Figure 3). Comparative structural analysis of the Tn*6218*-like element of KUB3006 suggested that it is almost identical to the Tn*6218*-like element present in *E. faecium* and *C. difficile* strains, rather than showing similarity to other *E. faecalis* Tn (Figure 3). This implies that the KUB3006, *E. faecium*, and *C. difficile* strains acquired the *cfr*(B)-positive Tn*6218*-like element from a common source. Moreover, the Tn*6218*-like element of KUB3006 did not perfectly match that of *E. faecalis* WH571, indicating that Tn*6218-*like elements in *Enterococcus* display variable Tn structures (Figure 3), although all of these elements carry *cfr*(B). Surprisingly, the Tn*6218*-like element of KUB3006 was 98.97% identical to the notable *C. difficile* Ox3196 strain isolated from a human in the United Kingdom in 2012 (Dingle et al., 2014) (Figure 3). The *C. difficile* BJ08 strain, which was isolated from a human in China in 2008, also carries a Tn*6218*-like element (GenBank ID: CP003939.1) identical to that of Ox3196, while *C. difficile* FDAARGOS_267 carries an element with the basic Tn structure without *cfr*(B).

**FIGURE 4:**
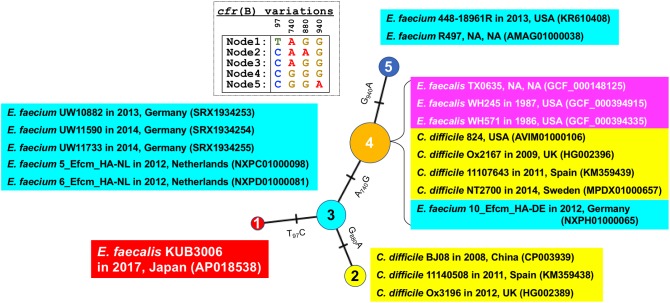
Median joining network analysis of the *cfr*(B) nucleotide sequence. Bacterial species, strain, isolation year, country and the GenBank ID (or assembly ID) are shown at the branch. Four SNV sites [97, 740, 880, and 940 nt position in *cfr*(B)] were highlighted as Table.

In addition, SNV analysis of the *cfr*(B) gene confirmed that the KUB3006 *cfr*(B) gene is more similar to those present in *E. faecium* strains isolated in EU countries from 2012 to 2014 than it is to other *E. faecalis cfr*(B) homologs (Figure 4).

### OptrA Ribosomal Protection Protein

A homology search for AMR genes revealed the LZD resistance gene *optrA*, which encodes an ABC-F subfamily ATP-binding cassette protein, on the plasmid pKUB3006-4 (36.3 kb) (Figure 1). pKUB3006-4 is identical in size and sequence (except for a 2-nt mismatch) to plasmid p6742_1 (GenBank ID: KY513280.1) of the LR *E. faecalis* strain 6742, which was isolated from a clinical pus specimen in 2012 in Poland (Gawryszewska et al., 2017). Polish LR *E. faecalis* strains primarily have G_2576_T 23S rRNA mutations and the additional plasmid-borne *optrA* gene but carry neither the *cfr* nor *cfr*(B) methyltransferases. Furthermore, pKUB3006-4 showed similarity to the pE394 plasmid (deposited as a partial sequence, GenBank ID: KP399637.1), which was previously identified in China in both clinical and livestock *E. faecalis* and *E. faecium* isolates (Wang et al., 2015), suggesting that this *optrA-*positive plasmid has been globally disseminated among *Enterococcus* species.

### Other Potential AMR Genes

In addition to *cfr*(B) and *optrA*, pKUB3006-1 (106.3 kb) carries multiple AMR genes, including *ant(6)-Ia, aph(3′)-III, dfrG, erm*(B), and *lnu*(B) (Figure 1). It has a similar backbone, with a 51% overlap, to the *vanA-*positive pTW9 plasmid in vancomycin-resistant *E. faecalis* (85.0 kb, GenBank ID: AB563188.1), which was isolated from poultry in Taiwan.

pKUB3006-2 (79.5 kb) carries no notable AMR genes (Figure 1) and has a similar backbone, with a 42% overlap, to the *E. faecalis* plasmid pGTC3 (GenBank ID: KY303941.1), which was isolated from the fecal material of a blue whale.

pKUB3006-3 (59.3 kb) carries multiple AMR genes, including *aac(6′)-aph(2′), erm*(B), *fosB3*, and *tet*(M) (Figure 1) and has a similar backbone, with a 52% overlap, to the *E. faecalis* plasmid pRE25 DNA (50.2 kb, GenBank ID: X92945.2), which was isolated from dry sausage in the EU (Schwarz et al., 2001).

## Discussion

In this study, we completed the whole-genome sequencing of an LR *E. faecalis* clinical isolate and revealed that this strain carries the notable *cfr*(B) 23S methyltransferase gene in a Tn*6218*-like element that is almost identical to a Tn from LR *E. faecium* and *C. difficile* strains. This is the first report of an *E. faecalis* isolate carrying a *cfr*(B)-associated Tn with a structural organization similar to that of a *C. difficile* Tn*6218*-like element. This structural comparison strongly suggests that *E. faecalis* KUB3006, *C. difficile*, and *E. faecium* may have acquired the Tn*6218*-like element under LZD treatment from a common source. Alternatively, this could represent a mutual horizontal Tn transfer between *Enterococcus* and *C. difficile* through phenicol selective pressure in a veterinary environment.

In general, the Tn*6218*-like elements in *C. difficile* are associated with a 19-kb pathogenicity locus (PaLoc) (Braun et al., 1996) that contains two large clostridial toxin genes (*tcdA* and *tcdB*) (Kuehne et al., 2010). The population structure of *C. difficile* consists of five clades based on PaLoc analysis (Dingle et al., 2014), and *C. difficile* Ox3196 is classified into PaLoc Clade 4. The *cfr*(B)-related Tn*6218*-like elements exhibit the variable acquisition of multiple AMR genes, including *cfr*(B), in a clade-independent manner (Dingle et al., 2014), suggesting that Tn*6218* elements occasionally contain genes conferring resistance to clinically relevant antibiotics in *C. difficile*.

In addition, analysis of *E. faecalis* KUB3006 revealed plasmids carrying multiple AMR genes, including *optrA*. Plasmid-mediated oxazolidinone resistance has been strongly linked to animal sources, in which the use of phenicols may co-select for resistance to both antibiotic families. Tamang et al. reported that in Korea, most LR *Enterococcus* isolates were also highly resistant to chloramphenicol and florfenicol, with no mutations in the 23S ribosomal RNA or in the ribosomal protein L3. In addition, these isolates did not carry *cfr* but were highly *optrA*-positive (Tamang et al., 2017). The *optrA* gene has been widely detected both in food-borne animals (poultry, pigs, and cattle) and clinical isolates in *E. faecalis* and *E. faecium*, whose STs belong to variable sequence types (Torres et al., 2018), indicating that *optrA* could be predominant resistance gene for LR *Enterococcus* species. Thus far, multiple *optrA* variants have been identified even in unrelated bacterial strains. Each *optrA* variant was located on the plasmids with the most identical background, indicating that the dissemination of *optrA* could be significantly involved in conjugative plasmid transfer (Bender et al., 2018). These observations suggest that KUB3006 may have initially acquired plasmid-mediated LZD resistance, followed by the acquisition of *cfr*(B).

Florfenicol is extensively used in livestock to prevent or cure bacterial infections. However, it is not known whether the administration of florfenicol has resulted in the emergence and dissemination of florfenicol resistance genes (FRGs, including *fexA, fexB, cfr, optrA, floR*, and *pexA*) in microbial populations in surrounding farm environments (Zhao et al., 2016). Zhao et al. (2016) detected FRGs and florfenicol residue in samples from six swine farms with a record of florfenicol usage. These authors concluded that the spreading of soils with swine waste could promote the prevalence and abundance of FRGs, including the LZD resistance genes *cfr, cfr*(B), and *optrA*.

Regarding the pathogenicity of *E. faecalis*, MLST analysis of EU strains indicated that multidrug resistance is common in the specific clonal complex (CC), in particular, the CC2, CC16, and CC87 lineages, whereas the CC2 and CC87 lineages were nearly exclusively observed in hospitals as potential “high-risk” *E. faecalis* lineages (Kawalec et al., 2007; Freitas et al., 2009; Willems et al., 2011; Kuch et al., 2012). However, KUB3006 is classified as ST729, which is a very minor ST. Considering that ST729 *E. faecalis* has not been reported previously, its complete genome sequence might uncover that KUB3006 carries multiple cell-adhesins, cell-damaging factors, sex pheromones, and aggregation substances (Table 2) that are characterized as pivotal virulence factors for infective endocarditis (Madsen et al., 2017). Although KUB3006 was isolated from a urine specimen of an immune-compromised patient with the successive infection, it might be a high virulent strain based on the identified set of *Enterococcal* virulence factors.

Potentially virulent *E. faecalis* KUB3006 strain harbors multiple LZD resistance determinants, *cfr*(B) and *optrA*, which contribute to LZD resistance (MIC: 16 μg/mL, Table 1). Indeed, the *cfr*(B)-positive *E. faecium* strains have been reported to exhibit an LZD MIC at 8 μg/mL (Deshpande et al., 2015), while *optrA*-positive *E. faecalis* strains have been reported to exhibit rather low MIC between 2 to 8 μg/mL (Torres et al., 2018). This suggests that KUB3006 might exhibit high MIC with multiple factors by *cfr*(B) and *optrA*, although the individual contribution of each gene remains to be investigated.

## Conclusion

The LR *E. faecalis* KUB3006 possesses a notable Tn*6218*-like-borne *cfr*(B) and plasmid-borne *optrA*, and this finding raises further concerns regarding the possible declining effectiveness of LZD treatment in the future.

## Data Availability Statement

The complete genomic sequences and annotations of *E. faecalis* strain KUB-3006 were deposited in a public database DDBJ: chromosome (AP018538); pKUB3006-1 (AP018539); pKUB3006-2 (AP018540); pKUB3006-3 (AP018541); and pKUB3006-4 (AP018542). The short- and long-read DNA sequences have been deposited in the DDBJ Sequence Read Archive under accession number DRA006641 (BioProject: PRJDB6823, BioSample: SAMD00113788-SAMD00113789, and Experiment: DRX11916-DRX119165).

## Author Contributions

KS, HS, and MS collected clinical specimens and isolated the strain from the patient. MK and TS performed the genome sequencing and the comparative genome analysis of *E. faecalis* KUB-3006. HM and HH contributed to the characterization of clinical isolates. MK wrote the manuscript.

## Conflict of Interest Statement

The authors declare that the research was conducted in the absence of any commercial or financial relationships that could be construed as a potential conflict of interest.
